# Identifying phase transitions in zeolitic imidazolate frameworks: microscopic insight from molecular simulations

**DOI:** 10.1039/d5sc09468b

**Published:** 2026-02-03

**Authors:** Léna Triestram, François-Xavier Coudert

**Affiliations:** a Chimie ParisTech, PSL University, CNRS, Institut de Recherche de Chimie Paris 75005 Paris France fx.coudert@chimieparistech.psl.eu

## Abstract

Metal–organic frameworks (MOFs) feature a rich structural diversity, including crystalline, amorphous, and liquid phases of varying topologies. Their structural characterization is often performed either at the local scale (through pair distribution functions, bond angle distributions, *etc.*) or, for crystalline phases, through topology analysis of the periodic framework—leaving out disordered and amorphous phases. In this work, we develop a computational methodology for the structural characterization of middle-range order in MOFs that is applicable to both crystalline and amorphous phases. We base our method on the statistical analysis of the geometry of the supramolecular framework at the microscopic level, and its evolution during molecular simulation. We analyze the statistics of metal–organic rings, their distribution in size, as well as their geometrical characteristics through mathematical tools derived from polymer physics: radius of gyration, asphericity, and writhe. We show that this advanced characterization can be leveraged for the identification of phases and the detection and analysis of phase transitions.

## Abstract 2

We present a computational methodology for the structural characterization of middle-range order in metal–organic frameworks (MOFs), based on analysis of their supramolecular rings and applicable to both crystalline and amorphous phases.

## Graphical abstract

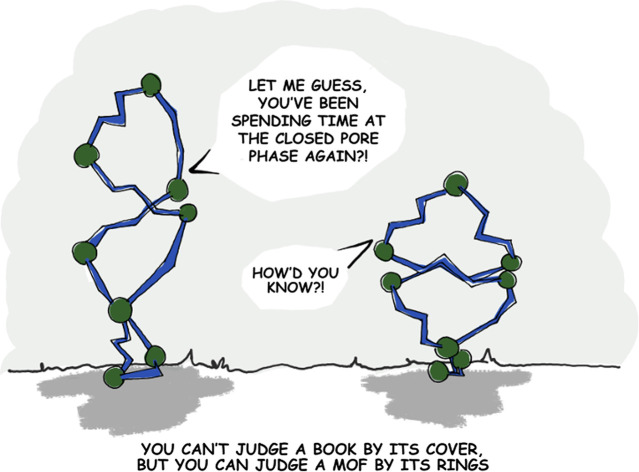

## Introduction

Metal–organic frameworks are a class of coordination polymers with limitless chemical versatility, due to the vast chemical space of their organic linkers and the larger number of possible metal centers and metal–ligand coordination modes. Alongside this chemical diversity, MOFs also display a wide variety of framework structure types, which can be identified by the analysis of the framework topology.^1–3^ Recent work on the CoRE MOF database, for example, has identified more than 340 different topologies out of 8857 published crystal structures,^4^ demonstrating the large structural diversity in MOF frameworks—a finding which can be generalized to other families of framework materials.^5^ Through the concept of reticular chemistry,^6^ researchers have explored the connection between the framework topology of a material and its physical properties,^7^ and to generate new structures of hypothetical MOFs with specific topologies^8,9^ and increased diversity in their topologies.^10^

However, MOF structures do not all necessarily differ in their topology, and different structures may share a topology, yet diverge widely in the microscopic arrangement of their atoms, as well as in the macroscopic properties arising from their structure. Moreover, MOFs can also exist as amorphous structures, in the liquid and glass state.^11,12^ Statistical characterization of these structures is of particular importance, but it is difficult to perform experimentally. Therefore, there is a need for the development of tools for the structural characterization of middle-range order in all phases of MOFs, applicable to both crystalline and amorphous phases.

**Fig. 1:**
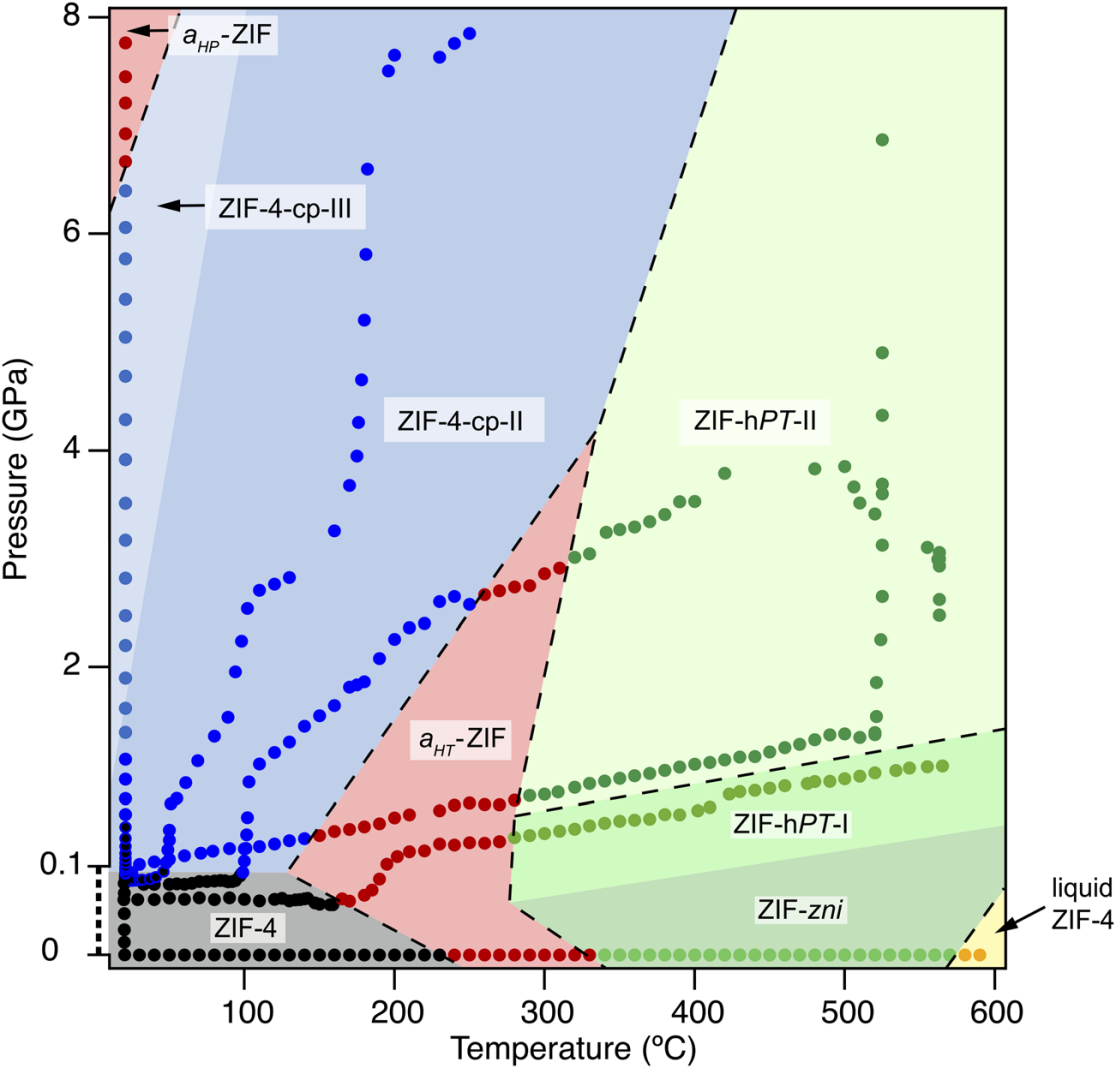
The phase diagram of ZIF-4 in temperature–pressure space, as put together by Widmer *et al.*^19^ from series of different high-temperature and high-pressure experiments. Reproduced from ref. 19 under the CC-BY license.

Among metal–organic frameworks, the subclass of ZIFs (or Zeolitic Imidazolate Frameworks)^13,14^ is particularly rich in chemical and structural diversity, featuring many possible functionalization of their linkers,^15^ polymorphism in both the crystalline and amorphous states,^16,17^ as well as stimuli-responsive behavior in the presence of guest molecules.^18^ The family of ZIF-4 (ref. 13) has been perhaps the most studied so far, presents several crystalline topologies as well as multiple amorphous phases reported experimentally, such as the high temperature glass, the high pressure glass and a liquid phase. Moreover, four isotopological phases have also been identified: ZIF-4, ZIF-4-cp, ZIF-4-cp-II and ZIF-4-cp-III (see in Fig. 1).^19^ Characterizing and identifying these phases is challenging, both experimentally and *in silico*, because several phases are structurally very close, as is the case of the closed pores phases, and several phase transitions do not involve any bond breaking.

In this work we develop and demonstrate a computational methodology for the structural characterization of middle-range order in MOFs, that is applicable to both crystalline and amorphous phases. We base our method on the statistical analysis of the geometry of the supramolecular framework at the microscopic level, and its evolution during molecular simulation. The method aims specifically at the identification of phases and the detection and analysis of phase transitions. Although our method is generic to all framework structures, we highlight it on the very challenging family of ZIFs, and we focus on phase transitions among ZIF-4 phases: crystalline, liquid and amorphous. While earlier work used ring statistics to compare different phases of ZIFs, our present work extends this approach to systematically analyze ring shape descriptors. It is thus able to distinguish in more details the different structures and phases, and unlike previous methods, it applies equally well to isotopological phases and their transitions.

## Systems & methods

We provide here a brief summary of the systems studied, and describe in detail the different computational methods used in this work. In order to make our work fully reproducible by others, representative input files for each type of simulation are available online in our data repository at https://github.com/fxcoudert/citable-data, as well as the data set of configurations generated through *ab initio* molecular dynamics (MD) and used for training and testing.

### ZIF-4

ZIF-4 has the chemical formula Zn(Im)_2_, where the Im ligands are imidazolate anions (C_3_H_3_N_2_^−^). In this structure, Zn^2+^ is 4-coordinated and the imidazolate linkers adopt a tetrahedral geometry around the metal. ZIF-4 has several known polymorphs with the same local arrangement around the metal but different topologies these are represented in the phase diagram of Fig. 1: ZIF-4 crystallises in the *cag* topology, and, other reported crystalline phases have distinct topologies, such as the denser ZIF-zni (*zni* topology) or ZIF-hPT-II (double-interpenetrated diamondoid network, *dia*).

ZIF-4 also has isotopological polymorphs such as the closed-pore phases, labelled “cp” they retain the same connectivity and differ by the shape of the pores: ZIF-4 is the open pore phase and ZIF-4-cp and ZIF-4-cp-II are the closed pore phases. These phases have been characterised experimentally and are accessible by increasing the pressure, starting from ZIF-4. In Fig. 1, the first closed-pore phase is not visited due to the high pressure increase rate: the ZIF-4 phase directly transitions to ZIF-4-cp-II. These isotopological phases are difficult to characterize in atomic simulation due to the similarity of their structures.

The last type of phase reported in Fig. 1 are amorphous phases, which are labeled a_HP_-ZIF and a_HT_-ZIF for the high-pressure and high-temperature glasses, respectively. A liquid phase is also present in the phase diagram. Being disordered, these phases do not have uniquely-defined topology—although work has been done to describe their network.^20^ To understand the differences between these phases, we need tools to characterize their structural properties.

This very rich polymorphism makes the ZIF-4 family a good playground to test new methodologies for phase identification: the isotopological phases cannot be distinguished by analyzing the topology of their network, and the amorphous structures are difficult to characterize and compare to other amorphous phases.

### Machine learning potential

To provide a chemically accurate description of the different phases of ZIF-4 for our molecular simulations, we trained a machine learning potential (MLP) using the MACE code^21,22^ (version 0.3.9). In order for this MLP to avoid bias towards known crystalline phases and be transferable across wide temperature and pressure ranges, we trained it on data sampled from the liquid phase of ZIF-4 at different values of temperature and volume (or density): *T* = 1500 or 1750 K, and volume change from the crystalline reference of Δ*V*/*V*_0_ = +2, 0, −2 or −4%. This data was obtained and characterized in previous work in our group,^23^ through *ab initio* molecular dynamics (AIMD) in the (*N*, *V*, *T*) ensemble of the ZIF-4 liquid.

The hyperparameters to train the model and the final mean absolute errors are detailed in Tables S2 and S3. The final model has a mean absolute errors of 1.43 meV per atom on the energy, 11.28 meV Å^−1^ on forces and 0.85 meV Å^−3^ on stress. Compared to our previous machine learning potentials based on nequip^24^ and Allegro^25^ and trained on the same data,^23^ this new MLP has a lower MAE on the prediction of atomic forces (11.28 meV Å^−1^, compared to 15.0 meV Å^−1^ and 30.1 meV Å^−1^), at the cost of a increase of the MAE on energy (1.43 meV per atom here, compared to 0.60 meV per atom and 0.70 meV per atom respectively).

Once trained, we validated this new MLP on structural properties such as the radial distribution function between Zn and N, the N–Zn–N angle distribution and the Zn–N potential of mean force of in ZIF-4, as well as the coordination number of the Zn atom of the glass and the liquid phase compared to *ab initio* MD in the (*N*, *V*, *T*) ensemble, we show these results in Fig. S1–S3 and Table S1 in the SI.

### Molecular dynamics simulations

The molecular dynamics simulations in this study were performed using LAMMPS^26^ (version 29 Aug 2024) using the LAMMPS_MACE model that relies on KOKKOS^27,28^ to offload calculations to the GPU—in this case, a NVIDIA Tesla V100. Simulations were run on a 2 × 2 × 2 supercell of ZIF-4 containing 2176 atoms, and employ a Nosé–Hoover thermostat and barostat with a damping factor of 0.1 ps for the thermostat and 1 ps for the barostat. The thermostat and barostat's equations of motion are those of ref. 29, and combine the hydrostatic equations of Martyna *et al.*^30^ with the standard strain energy from the Parrinello–Rahman scheme.^31^ Prior to production simulations and data gathering, the structures were equilibrated at 300 K in the corresponding ensemble.

For the melt–quench simulation, we followed a similar protocol to the protocol reported in ref. 23: we performed the melt–quench simulation in the (*N*, *V*, *T*) ensemble, with a timestep of 0.25 fs. We used the DFT-optimised structure with fixed cell from Widmer *et al.*^19^ as a starting point, which was first equilibrated at 300 K. After that equilibration, a series of temperature ramps were performed to obtain the liquid phase: 5 ramps and 5 plateaux were employed from 300 K to 1500 K, each of them lasting 5 ps. The temperature was then maintained at 1500 K for 230 ps, and finally the liquid was quenched to 300 K in a procedure similar to the melting process. The processes observed are not sensitive to the exact melt–quench protocol followed, but the use of several ramps and plateaux allows us to perform statistical analysis of the structures at well-defined intermediate temperatures.

The simulations to generate high-pressure phases from ZIF-4 were performed in the (*N*, *P*, *T*) ensemble with a timestep of 0.5 fs. We used 5 ramps and 5 plateaux to bring the simulation from the pressure of equilibrium of ZIF-4 (which is 0.65 GPa with this model) to the pressure of equilibrium of ZIF-4-cp-II (1.4 GPa). These pressures are higher than the reported experimental pressures^19^ (respectively 0 GPa and 0.65 GPa for ZIF-4 and ZIF-4-cp-II at 25 °C), as general shift which leads to qualitative, but not quantitative agreement in pressure values. This issue finds its root in the fact the MLP does not reproduce well the density in experimental conditions (or, seen in another way, the pressure at fixed density)—and the issue is rather general, because it was previously reported for another MLP on the same material.^32^

Finally, we also performed MD simulations in the (*N*, *P*, *T*) ensemble at varying pressures for ZIF-4, ZIF-4-cp and ZIF-4-cp-II, again with a timestep of 0.5 fs. We used the ZIF-4-cp structure from Méndez *et al.*^33^ and ZIF-4-cp-II fixed-cell structure from Widmer *et al.*^19^ as starting points for the simulations.

### Identification of rings

**Fig. 2:**
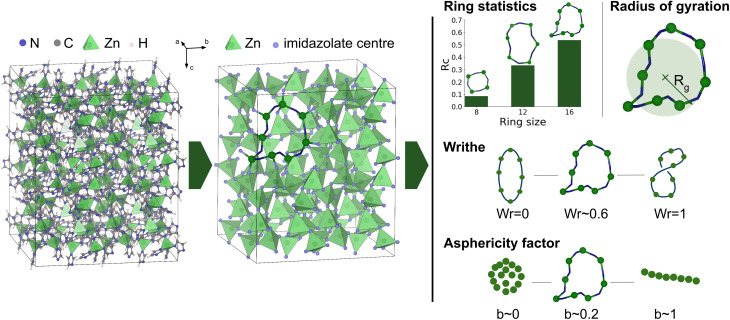
Workflow of the methodology for identification and characterization of zinc–imidazolate rings, exemplified on the structure of the ZIF-4 crystal. (i) The imidazolate linkers are first reduced to their center of mass. (ii) From this structure, irreducible rings are identified. (iii) Rings can be analyzed in different ways: statistics of ring sizes, and characterization of their geometry using different tools, namely the radius of gyration, writhe and asphericity factor. Visualization of the atomic structures is performed with VESTA.^34^

Rings are defined as closed paths made by alternations of the metal ions (Zn^2+^) and imidazolate linkers. To identify them from the MD trajectories, we first simplify the structure by coarse-graining the imidazolates to a single point, as shown in Fig. 2. In this simplified structure, rings are calculated between the center of the ligands and the Zn atoms with a cutoff distance of 4 Å (based on the Zn–N radial distribution function). Reducing each ligand to a single point allows us to reduce computational cost of ring identification, and more intuitive analysis of the rings and their shapes (abstracting the detailed motion of all atoms in the ligands).

From the coarse-grained structure, we calculate rings with the RINGS code^35^ using the *primitive rings* criterion,^36,37^ also referred to as *irreducible ring*:^38^ such rings cannot be decomposed into two smaller rings.

We number the rings by counting the number of both metal and linker entities. For example, 16-membered rings are constituted of 8 Zn atoms and 8 imidazolate linkers. To calculate the ring statistics, we use the number of rings per cell *R*_c_. This quantity is calculated by counting the number of rings of a specific size and dividing by the total number of nodes (total number of Zn and imidazolates).

We additionally calculate the coordination number of the Zn metal, which is computed with a 2.5 Å cutoff between the Zn and N atoms.

### Radius of gyration, asphericity and writhe

We present in this section the mathematical tools we have applied to analyze the shapes of the rings identified above. We have used three different geometric markers to track structural changes during simulations: the radius of gyration, writhe, and asphericity factor.

The *radius of gyration R*_g_ describes the average radius of a set of points from its center of mass, *i.e.*, it is the second moment of mass distribution of an object.^39^ It is given by:1
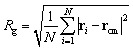
where **r**_*i*_ is the position of the *i*^th^ atom, and **r**_cm_ is the center of mass of the ring—in turn calculated as 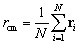. While it is frequently used in polymer physics, where it is a key quantity in the description of polymer chains and the statical models that govern them,^40^ in this study we use *R*_g_ to describe the isotropic extension (or contraction) of the rings in space.

The second descriptor we will use, on the other hand, relates directly to the anisotropy of the geometry of the rings. The gyration tensor **Q** is a 3 × 3 matrix which describes the spacial distribution of the *N* atoms of a ring along directions *α* and *β*. It is the tensorial form of the second moment of the mass distribution of our “cloud of points”, defined as:2
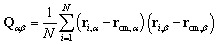


From the eigenvalues {*λ*_1_, *λ*_2_, *λ*_3_} of **Q**, we can obtain the *asphericity factor*^41,42^ (noted *b*) through:3
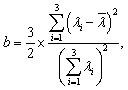
where *λ̄* is the mean of the eigenvalues. The asphericity factor is a quantity between 0 and 1: a value close to 0 describes a set of points arranged spherically in space (an isotropic distribution), while a value close to 1 describes a very elongated shape.

Finally, the *writhe* (Wr) describes how much a given ring crosses over itself. We use pyknotid^43^ (version 0.5.3) to calculate the writhe of the rings by numerically approximating the writhe integral:4
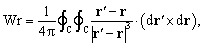
where *r* and *r*′ are two points on the curve *C*. This value can be positive or negative, depending on the crossing direction (following the right hand rule). As represented in Fig. 2, if the ring is completely flat, the writhe is equal to zero; and if the ring crosses over itself once, it is equal to ±1.

### Smooth overlap of atomic positions (SOAP) & t-SNE

As a complement to the structural analysis performed through rings characterization, we also used the local Smooth Overlap of Atomic Positions (SOAP) descriptor^44^ on the Zn centers. SOAPs provide translation, rotation and permutation-invariant descriptors of groups of atoms, and are often used to describe the local environment of an atom and represent it as a vector.^45^ Here, we use them to compare the local environments of several Zn atoms in structures belonging to different phases, and to track phase transitions along molecular dynamics trajectories.

To calculate the SOAPs, we used the DScribe library^46,47^ (version 2.1.1) with a 9 Å cutoff, 12 radial basis functions and a degree of spherical harmonics up to 9. The functions that represent the atoms are Gaussians with a standard deviation of 0.3, and the radial basis functions are spherical Gaussian type orbitals. To be able to project this multidimensional vector in 2 dimensions for the purpose of visualization, we used t-distributed Stochastic Neighbor Embedding (t-SNE).^48^ We calculated the t-SNE with the scikit-learn package^49^ (version 1.6.1).

## Results & discussion

### Ring statistics to characterize amorphous phases

One common way to describe the structure of crystalline phases of framework materials in general,^5^ and MOFs in particular,^50^ is by identifying the topology formed by their network. However, analysis of the global framework topology is not applicable to amorphous phases, disordered crystalline structures,^51^ and does not distinguish between crystalline phases (or polymorphs) of identical topology—something that is particularly common among MOF materials^19,52^ and soft porous crystals.^53^

In order to statistically analyze supramolecular frameworks, it is also possible to study the statistical distributions of the rings which compose their structures, shedding light on their connectivity. For typical MOFs, these rings are formed by alternations of metal centers and organic ligands, forming a closed loop. The analysis of ring size distributions (*i.e.*, the statistical distribution of rings of different sizes in the frameworks) has been reported in the literature on disordered and amorphous phases of MOFs in the past, allowing to distinguish phases and quantify structural changes. For example, ring statistics were used to describe the nucleation processes at play in the early stages of self-assembly of ZIF-8.^54^ They have also been used to characterize the high-temperature glass phase of ZIF-4 (ref. 23) and in a later work to find a quantitative measure of the topology of this phase in order to compare it to the networks formed in inorganic glasses.^20^

Going beyond such static analyses, we demonstrate in the present work how a dynamic analysis of ring statistics over time in a molecular dynamics trajectory can be used to characterize phase transitions involving bond breaking and forming (*i.e.*, changes of topology of the framework). We first use the ZIF-4 → liquid → glass transitions as an example, following an *in silico* melt–quench protocol. The ZIF-4 crystal is heated from 300 K to 1500 K, followed by a plateau at 1500 K and the temperature is then brought back to 300 K to form the glass. A similar protocol has been used in previous studies in our group^23,55^ to generate glass through *ab initio* molecular dynamics, and it has been shown that 1500 K is above the melting point.^19^

**Fig. 3:**
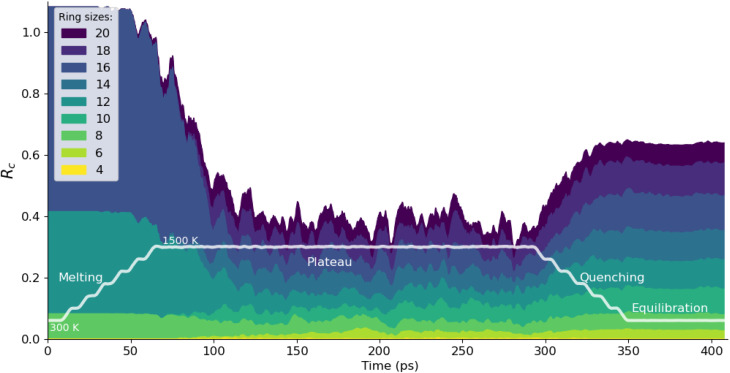
Evolution of ring statistics during a melt–quench simulation starting from crystalline ZIF-4, in the (*N*, *V*, *T*) ensemble (the curve has been smoothed with a moving average of 10 configurations).

As shown in Fig. 3, the network formed by crystalline ZIF-4 has perfectly well-defined rings: 8, 12, and 16-membered rings, with ring counts of *R*_c_ = 0.08, 0.33 and 0.67 respectively (consistent with previous experimental and theoretical studies^19,23^). Upon heating, the total number of rings decreases, and the variety of ring types increases; we calculated and report ring sizes ranging from 4 to 20.††Larger rings exist but in very small amounts, and the computational effort for ring detection increases drastically fast with maximal ring size. This is accompanied by a decrease in coordination number for the zinc: the ZIF-4 Zn has a coordination number of 4, and as the liquid forms, this value decreases to 3.54. Comparable values have been reported for *ab initio* MD (3.52) and using another MLP (3.67).^23^ In this liquid phase, we see that rings are formed and broken over time, with a statistical distribution that reflects the strongly associated nature of the liquid, with local tetrahedral ordering, as in liquid silica^56^ and water.^57^

During the quenching process, an increase in the total number of rings in the structure reveals the formation of the glass, which goes along with an increase of the coordination number to 3.92 (again in line with previous data from *ab initio* MD at 3.93,^58^ see Table S1). During the quenching process, the diversity of the ring types is, however maintained. We see the introduction of multiple ring sizes, which are not present in the original crystalline ZIF-4 structure, however, some of these ring sizes are present and stable in other crystalline polymorphs such as ZIF-hPT-II (12 and 20-membered rings) and ZIF-zni (8, 12 and 24-membered rings). The distribution of ring sizes and the number of rings in the glass phase are good indicators of the medium-range order in its atomic structure, although there is relatively little experimental data for validation.

### Ring shapes to characterize isotopological frameworks

Ring size distributions provide a valuable tool to characterize transitions that exhibit bond breaking: the change in the number and size of rings is due to a change in the metal–ligand coordination. This means that we need different tools to identify phase transitions in structures that share the same topology, focusing not only on the distribution of sizes, but on their shapes. In ZIF-4, an *ad hoc* approach along these lines was reported by Méndez *et al.*, calculating simple quantities such as the distance between two ligands of 16-membered rings.^33^

In this work, we propose a systematic methodology that relies on statistical analysis of the shape of rings, in order to study transitions between isotopological phases. The tools presented here have been used in past studies in the fields of polymer physics^59^ and DNA supercoiling.^60^ This approach has also been used in the case of polyamorphism to distinguish two hypothetical liquid phases of water by the topology of their hydrogen bonds network.^61^

To do so, we combine three different markers of rings shape: the radius of gyration, the asphericity factor and the writhe (depicted schematically on Fig. 2, and whose mathematical definitions are provided in the Methods section). All three features can be quantitatively compared across different phases, and are easily interpretable as they describe specific and intuitive geometrical characteristics. For example, comparing two closed loops, one can ask: “what is the size of each loop?” (radius of gyration); “how much do they coil around themselves?” (writhe); “is the shape rather spherical or elongated?” (asphericity).

We use this method to analyze isotopological phase transitions in the rich system of ZIF-4 polymorphs: in other words, phase transitions that cannot be probed by calculating ring statistics, because they do not involve bond breaking or formation. In order to highlight the method, we focus on the ZIF-4 → ZIF-4-cp → ZIF-4-cp-II phase transitions as illustrative examples.

### Open-pores to closed-pores transition

We first aim to show these computed quantities for two phases, ZIF-4 and ZIF-4-cp-II, in order to understand the nature of their distributions and link them to the behavior of individual rings in the structures. These distributions can then be used as a reference when looking at phase transitions. To characterize ZIF-4 and ZIF-4-cp-II, we first look at 16-membered rings because they are present in high quantity (*R*_c_ = 0.67) and are the largest interpretable rings, allowing for more flexibility. This is important as we aim to see a change between different phases.

**Fig. 4:**
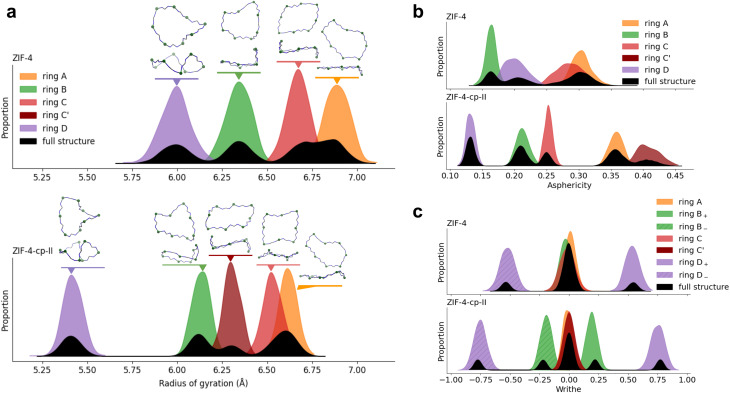
The radius of gyration (panel a), asphericity factor (panel b) and writhe (panel c) distributions of 16-membered rings in ZIF-4 and ZIF-4-cp-II. In panel (a), individual rings are identified in each structure that correspond to the different modes of the distributions.

Fig. 4 shows the distribution of the radius of gyration, asphericity factor and writhe for both phases. We also break these distributions into contributions from crystallographically distinct rings, which we label from A to D, and for which we provide snapshots highlighting their typical shape. Within a phase, the shape of individual rings did not change drastically during the MD simulations, and the distribution of each ring's characteristics has a Gaussian profile. We also confirmed that the distributions found with our MACE MLP for a 2 × 2 × 2 supercell are comparable to the results of the *ab initio* MD for a single cell, as shown in Fig. S4—these results reflect the framework structures, and are not model-dependent.

Comparing the results for the two phases, it is clear on Fig. 4 that the distributions of all three characteristics (radius, asphericity and writhe) are clearly distinct between the parent ZIF-4 and its cp-II phase. ZIF-4-cp-II is a higher-pressure phase, which is reflected in the shape of the rings as they contract, *i.e.*, the radius of gyration decreases for all rings (Fig. 4a). With increased pressure, the writhe of the D ring increases, and the B ring acquires a nonzero writhe. Note that in Fig. 4c, we label rings with a negative writhe *X*_−_ and rings with a positive writhe *X*_+_. The sign of the writhe refers to the convention of the crossing direction. The *X*_+_ and *X*_−_ rings have the same asphericity and radius of gyration, they are enantiomers.

Depending on how the ring contracts, the asphericity changes differently: for example, in the C ring of ZIF-4, the asphericity either decreases when contracted in one direction (C-ring of cp-II) or increases when contracted in another direction (C′ of cp-II). We find that the parent ZIF-4 is more symmetric than the cp-II phase, where the transition splits equivalent rings into two distinct populations. We can see this with the writhe of the B ring, which goes from a writhe distribution centered around 0, to ±0.25. It is also noticeable with the C ring, which splits its behavior in ZIF-4-cp-II.

### Variation of ring characteristics in a single phase

These distributions of radius, asphericity and writhe being unique to each phase, we can use them as a reference to characterize and identify the various polymorphs of identical topology in molecular dynamics simulations. Before we do that, it is important to test the robustness of the method, we calculated these quantities for the ZIF-4 parent phase at different pressures. Looking at the same phase in different conditions helps us examine whether the shapes of the rings differ only for different phases or whether they can differ within a given phase. By doing this, we make sure that these tools are able to pick up true phase transitions and not slight differences in atomic environments.

**Fig. 5:**
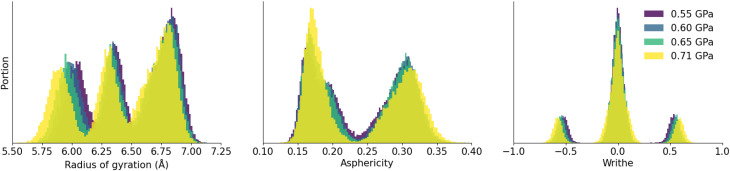
Distributions of radius of gyration, asphericity and writhe of 16-membered rings in ZIF-4 at different pressures, from molecular dynamics trajectories in the (*N*, *P*, *T*) ensemble at *T* = 300 K.

In Fig. 5, we show the features which characterize the shapes of 16-membered rings in ZIF-4 at different pressures ranging from 0.55 to 0.71 GPa. The distributions of these quantities do not change abruptly with pressure, and the characteristic peaks of ZIF-4 remain unchanged for the radius of gyration, asphericity factor and writhe. The increase in pressure is followed by a small decrease in gyration radius and increase in absolute writhe, which is in line with chemical intuition. The asphericity slightly increases for the peak centered on 0.3 and slightly decreases for the peak centered on 0.2. We also report in Fig. S5 a comparison of these quantities between simulations performed in the (*N*, *P*, *T*) and (*N*, *V*, *T*) ensembles, and find consistent results in both ensembles.

The distributions of writhe, asphericity factor and radius of gyration are different for each phase and, for a given phase, only slightly depend on the pressure of the simulation or the methodology: they are unique fingerprints of the open-pore and closed-pore systems and can help us identify phases during a simulation.

### Analysis of MD trajectories

We now analyze a trajectory in which we vary the pressure from the pressure of equilibrium of ZIF-4 to the pressure of equilibrium of ZIF-4-cp-II and track the evolution of the radius of gyration, asphericity and writhe. The evolution of density over the trajectory is depicted on Fig. S6. For clarity, we focus on the ZIF-4 → ZIF-4-cp-II transition, however, the transition is reversible and the opposite transition has also been simulated with similar findings (see Fig. S7 for details).

**Fig. 6:**
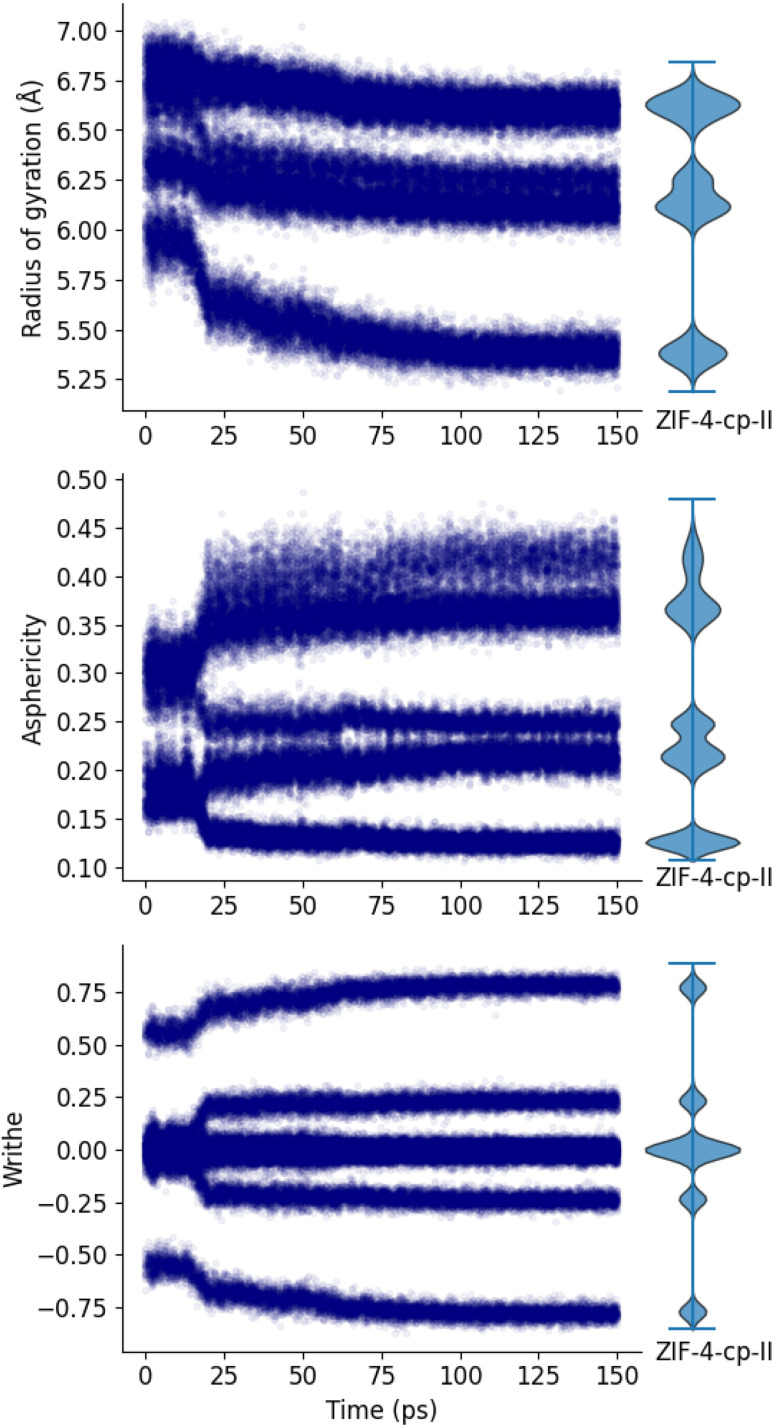
The three markers (radius of gyration, asphericity factor and writhe) describe the shape of 16-membered rings, these are used to characterize the phase transition between ZIF-4 and the closed-pores phases.

Fig. 6 shows that our three markers are good tools to differentiate between the open pore and the closed pore systems: we can see an abrupt change in each marker at *t* = 17 ps which shows the transition between the ZIF-4 phase and the closed pore phases. The corresponding pressure is 0.75 GPa. Using the shape of 16-membered rings, it is however, not possible to differentiate between the two closed pore phases (ZIF-4-cp and ZIF-4-cp-II). To compare the shape of the final rings to the expected phase, we also plot the distributions of the ZIF-4-cp-II phase in Fig. 6, as violin plots on the right. The final distributions of each of these markers in our MD trajectory correspond to those determined for ZIF-4-cp-II. This can be used to confirm that the phase we obtain at the end of the simulation is truly the one expected, and that the MLP force field (trained on the high-temperature liquid phase) can suitably describe phase transitions in the ZIF-4 system.

### Transition between two closed-pores phases: ZIF-4-cp and ZIF-4-cp-II

Analyzing 16-membered rings is useful to characterize the transition between open-pore and closed-pore systems. However, they do not allow for differentiation between the two closed pore phases due to the similarities between these two structures. There, we can rely on the analysis of smaller rings. The 12-membered rings are less flexible than the previously analyzed 16-membered rings. The change of the markers is therefore smaller, however, they can be used to differentiate between the ZIF-4-cp and ZIF-4-cp-II phases. Other ring sizes have been used previously as well: for example to differentiate ZIF-4-cp and ZIF-4-cp-II, a study had analyzed 8-membered rings, looking at the orientation of these rings with respect to the cell vectors.^33^

**Fig. 7:**
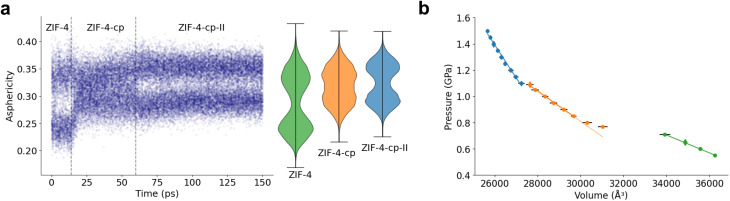
(a) The change in the distribution of the asphericity factor of 12-membered rings compared to the asphericity distribution in each phase. (b) Pressure as a function of volume for several simulations in the (*N*, *P*, *T*) ensemble.

Fig. 7a shows the evolution of the asphericity of 12-membered rings as a function of MD time, and compares them to the reference asphericity distributions of the three phases considered. As pressure increases, the asphericity of 12-membered rings changes from the distribution of ZIF-4 to that of ZIF-4-cp, and eventually to ZIF-4-cp-II at about 1.1 GPa. Following the asphericity over time allows us to see clear phase transitions characterized by a sudden jump in the distributions. We also provide the radius of gyration and writhe of the 12 and 8-membered rings and the asphericity factor of the 8-membered rings in Fig. S8 and S9.

To further confirm the presence of ZIF-4-cp in this simulation and test the robustness of this method to characterize phases, we also plot the pressure as a function volume for several simulations in Fig. 7b. These values come from the plateau in the simulations of ZIF-4 → ZIF-4-cp-II and additional simulations in the (*N*, *P*, *T*) ensemble. We can clearly identify the different phases by their different slopes (three different linear regimes) and the occurrence of phase transitions, matching the structural observations above.

**Fig. 8:**
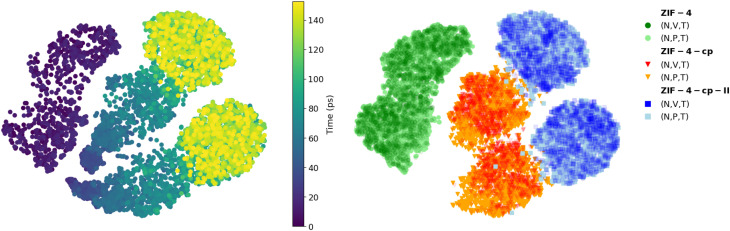
t-SNE of the Zn-centered Smooth Overlap of Atomic Positions (SOAP) descriptors for the ZIF-4 → ZIF-4-cp-II simulation (left panel) and for each individual phase in both the (*N*, *V*, *T*) and (*N*, *P*, *T*) ensemble (right panel).

Finally, we also calculated the local SOAP descriptor of all Zn atoms during the simulation and compare it to the SOAP of the known phases using the t-SNE dimensionality reduction technique. The results, plotted on Fig. 8, show how the SOAP changes over the simulation (left panel). We compare this evolution to the distribution of the reference SOAP values of each phase simulated individually (right panel), in both (*N*, *V*, *T*) and (*N*, *P*, *T*) conditions. We can see that at the beginning of the MD trajectory (points in dark purple), the local environments are the same as the environments in ZIF-4. There is a gradual evolution as pressure increases, followed by a sudden transition (in cyan) to a distinctive region of the t-SNE plot, with SOAPs comparable to ZIF-4-cp phase (right panel, orange). A second transition (green and yellow) leads to the ZIF-4-cp-II phase, again in a distinct region of t-SNE space (blue in the right panel).

## Conclusions & perspectives

We have developed a computational methodology for the structural characterization of middle-range order in MOFs that is applicable to both crystalline and amorphous phases. While we see that generic geometric atom-based descriptors, such as SOAPs (and their reduced dimensionality versions through t-SNE) can characterize different phases of MOFs, such results lack physical interpretability and do not provide chemical insight into the differences between phases. Therefore, we proposed physical meaningful methods to identify phases in a ZIF family of materials, based on the rings of its framework. These methods were used to characterize phase transitions between different polymorphs of ZIF-4. Two main approaches were presented: in the case of transitions that involve ligand rearrangement, ring statistics can be used to track structural changes during the simulation. It was then possible to follow the transition from a crystal structure to an amorphous structure, as well as between two amorphous structures. The other approach was used for isotopological phase transitions: when the transition does not feature bond breaking, the number of rings remains unchanged, as it is the case in the ZIF-4 → ZIF-4-cp → ZIF-4-cp-II transitions. In this case, we can characterize the phase transitions by looking at the ring's shapes. To do so, we used three markers: the radius of gyration, the asphericity factor and the writhe to quantify the change in the ring's shapes that occur during the transition.

In this paper, we have applied these new methods of structural characterization to the ZIF family, because they have the most complex phase diagram of any MOF in the literature. However, our methods are entirely generic, being based on the decomposition of the framework into building units and the analysis of primitive rings in this framework—regardless of the computational level of theory used, or choice of force field, or other methodological details. Therefore, it can be applied to any given MOF structure once the secondary building units are defined, *i.e.*, once a choice of clustering of the structure is performed. For MOFs with more complex SBUs, different type of linkers or defects, the methodology can be adapted on a case to case basis. Moreover, the method is also applicable to other framework materials, including covalent organic frameworks, supramolecular organic frameworks, and inorganic framework materials. We have even shown that the analysis of rings is valuable in the liquid phase, and could be applied to other strongly associated liquids, or solid–liquid transitions.

Moreover, this type of geometrical analysis for phase identification could be performed in a more local fashion. In future work, we intend to focus on molecular simulations of MOF phase transitions at much larger length scales, allowing us to study the dynamics of the transitions and perform spatial analysis of the transitions. This will allow us to use our geometry-based tools in order to answer questions such as: how does the transition proceed over time? What is the nucleation and growth process for the target phase? Is there possibility of phase coexistence, and if so on what characteristics length and time scales?

## Author contributions

L. T.: conceptualization, formal analysis, investigation, methodology, software, visualization, writing – original draft, writing – review & editing. F.-X. C.: conceptualization, methodology, supervision, writing – review & editing.

## Conflicts of interest

There are no conflicts to declare.

## Supplementary Material

SC-OLF-D5SC09468B-s001

## Data Availability

In order to make our work fully reproducible by others, representative input files for each type of simulation are available online in our data repository at https://github.com/fxcoudert/citable-data, as well as the data set of configurations generated through *ab initio* MD and used for training and testing of the machine learning potential. Supplementary information (SI): values of MLP hyperparameters, metrics of the final model, additional structural characterization. See DOI: https://doi.org/10.1039/d5sc09468b.
