# Supplementary material for: Identifying phase transitions in zeolitic imidazolate frameworks: microscopic insight from molecular simulations
Source: Chem Sci. 2026 Feb 3;17(13):6734–45. doi: 10.1039/d5sc09468b (PMC12893120; doi:10.1039/d5sc09468b)
Supplement: SC-017-D5SC09468B-s001 [file SC-017-D5SC09468B-s001.pdf]

Supplementary Information (SI) for Chemical Science.
This journal is © The Royal Society of Chemistry 2026

Supporting Information for: Identifying Phase Transitions in Zeolitic Imidazolate Frameworks: Microscopic Insight from Molecular Simulations

Léna Triestram and François-Xavier Coudert*

Chimie ParisTech, PSL University, CNRS, Institut de Recherche de Chimie Paris, 75005 Paris, France

E-mail: fx.coudert@chimieparistech.psl.eu

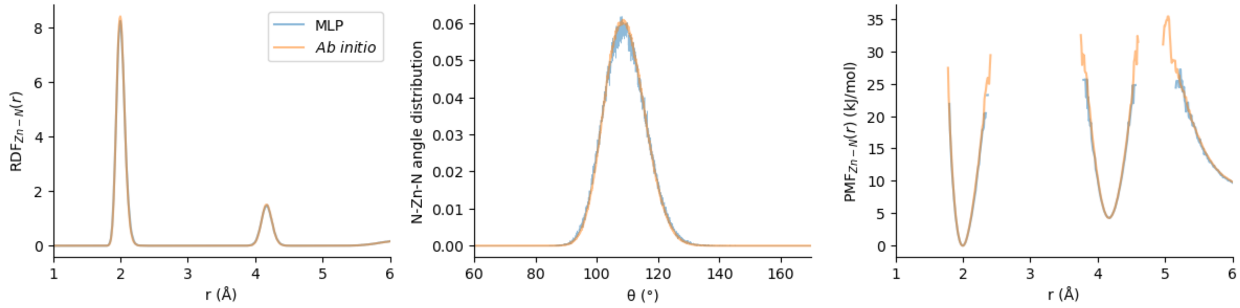

Figure S1: Comparison of structural properties between simulations performed with the MLP and AIMD for ZIF-4 in the (N, V, T) ensemble: Zn–N radial distribution function, N–Zn–N angle distribution and Zn–N potential of mean force.

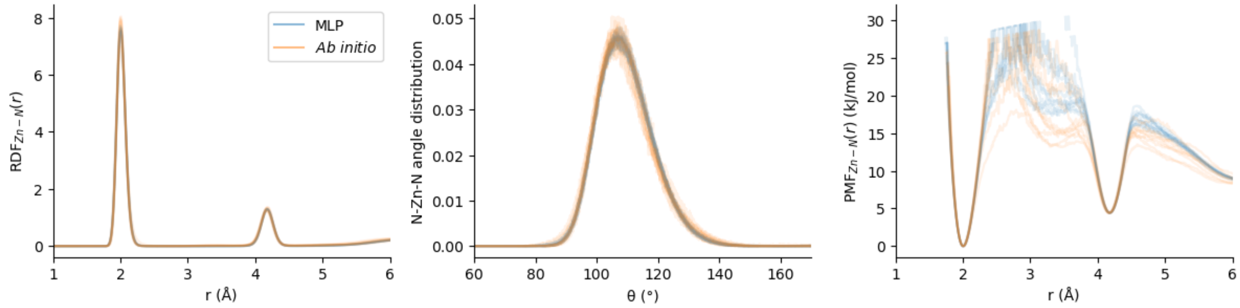

Figure S2: Comparison of structural properties between simulations performed with the MLP and AIMD for 10 different glasses in the (N, V, T) ensemble: Zn–N radial distribution function, N–Zn–N angle distribution and Zn–N potential of mean force.

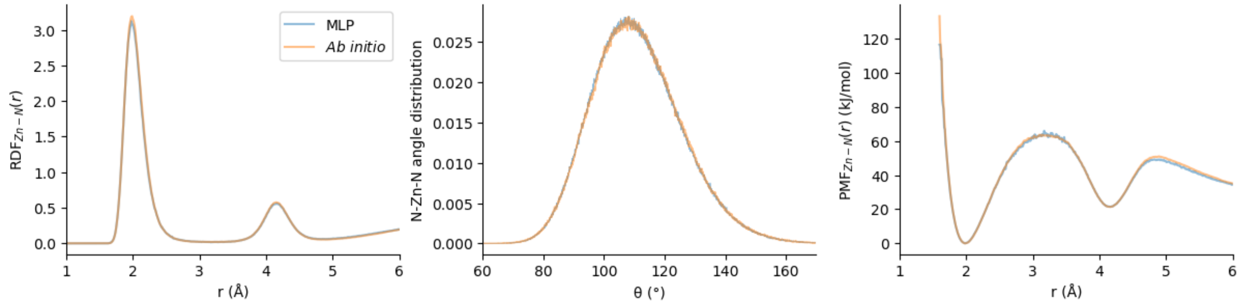

Figure S3: Comparison of structural properties between simulations performed with the MLP and AIMD for the liquid phase in the (N, V, T) ensemble: Zn–N radial distribution function, N–Zn–N angle distribution and Zn–N potential of mean force.

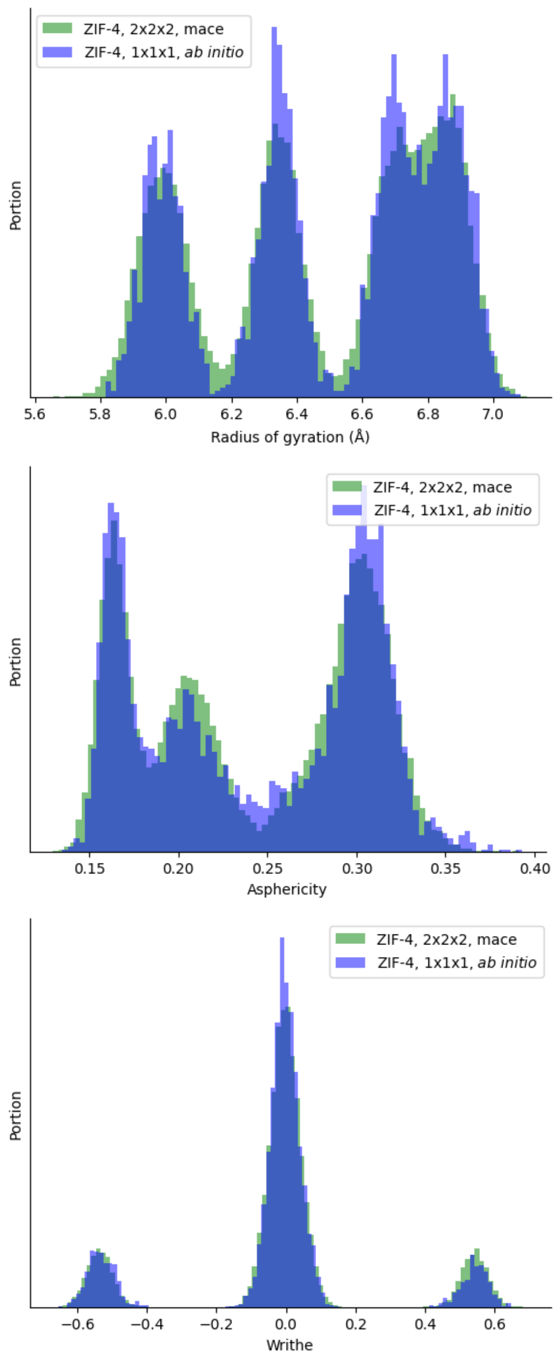

Figure S4: The radius of gyration, asphericity factor and writhe distributions of 16-membered rings in the (N, V, T) ensemble simulated on a supercell using the MACE MLP and on a single cell with *ab initio* MD.

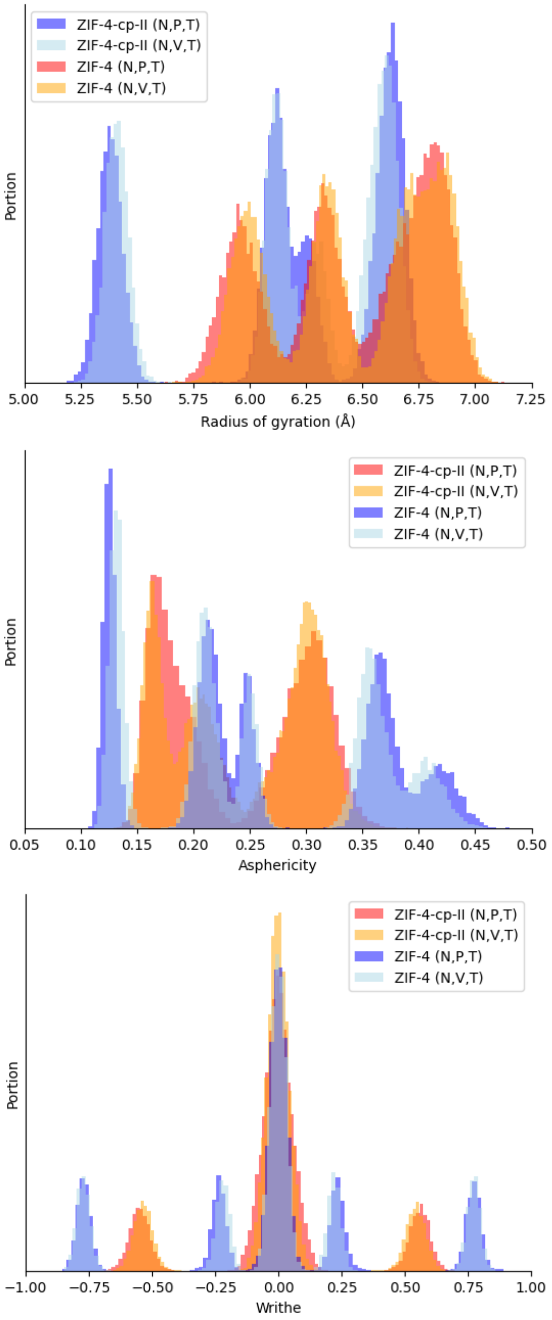

Figure S5: Comparison of the radius of gyration, asphericity factor and writhe distributions of 16-membered rings for ZIF-4 and ZIF-4-cp-II between the (N, V, T) and (N, P, T) ensembles.

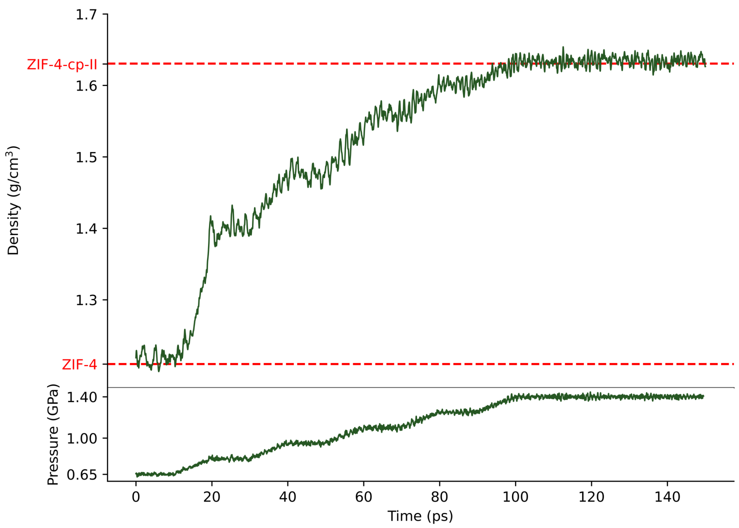

Figure S6: Change in density in the (N, P, T) simulation for the ZIF-4 $\rightarrow$ ZIF-4-cp-II simulation. The pressure is given at each time. The transition from ZIF-4 to ZIF-4-cp occurs around 0.75 GPa, and from ZIF-4-cp to ZIF-4-cp-II around 1.1 GPa.

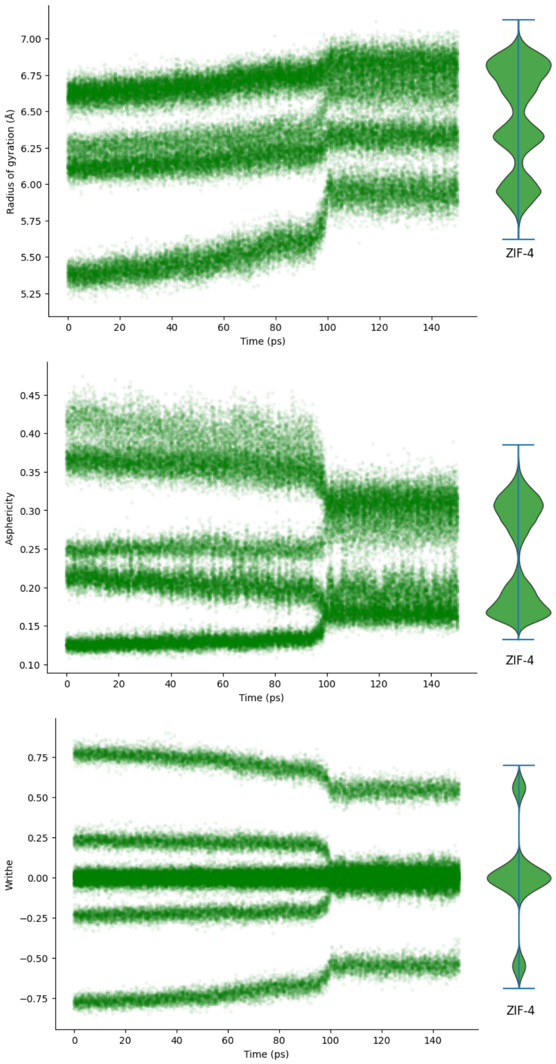

Figure S7: The radius of gyration, asphericity factor and writhe distributions over time for the ZIF-4-cp-II → ZIF-4 simulation in 16-membered rings.

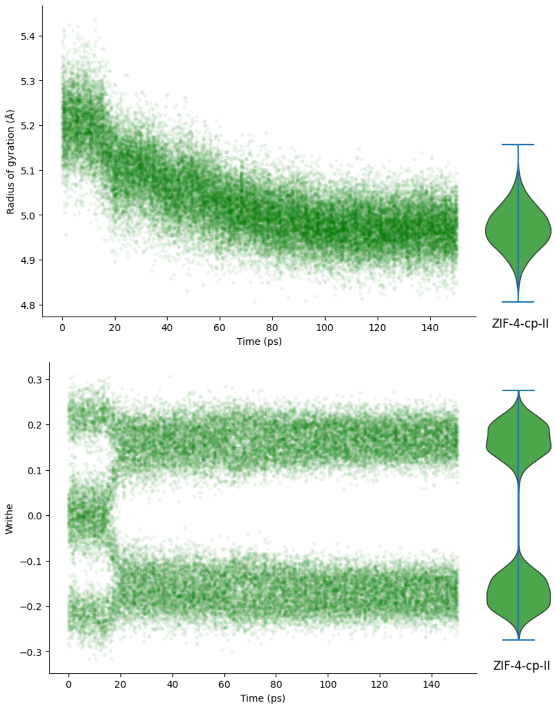

Figure S8: The radius of gyration and writhe distributions over time for the ZIF-4 → ZIF-4-cp-II simulation in 12-membered rings.

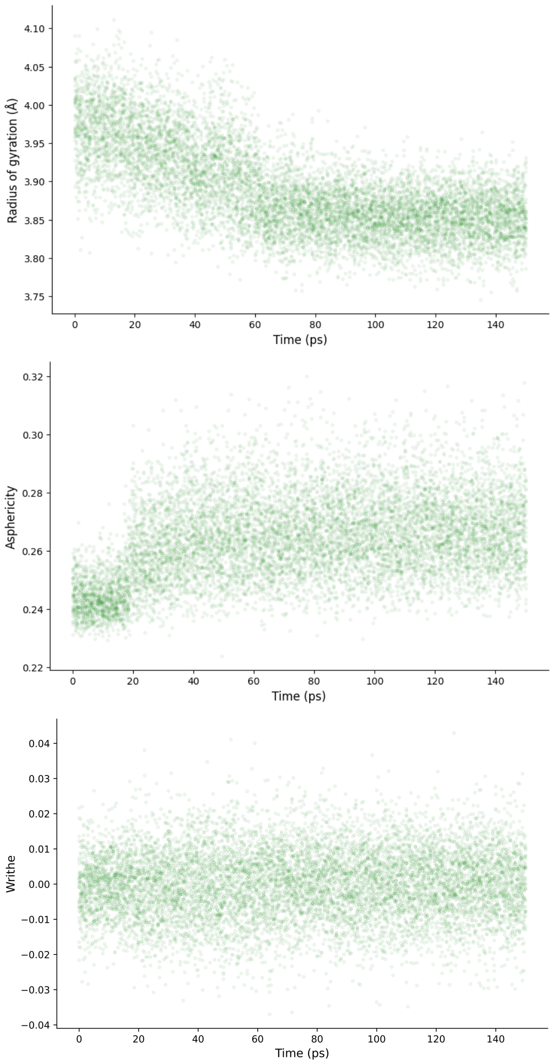

Figure S9: The radius of gyration, asphericity factor and writhe distributions over time for the ZIF-4 $\rightarrow$ ZIF-4-cp-II simulation in 8-membered rings.

Table S1: Zn–N coordination numbers for ZIF-4, the liquid and the glass phases.

coordination number	ZIF-4	glass	liquid
ab initio	4	3.93	3.52
MLP	4	3.92	3.54

Table S2: Hyperparameters used for the training of the MACE MLP.

Hyperparameters	Value
num_channels	128
max_L	1
max_ell	3
num_interactions	2
correlation	3
r_max	6.0
energy_weight	1
forces_weight	10
stress_weight	100
ema_decay	0.99

Table S3: Mean Absolute Errors (MAE) of the MLP.

Energy	1.43 meV/atom
Forces	11.28 meV/Å
Stress	0.85 meV/Å^3/atom
